# Exploring the professional quality of life of South African audiologists

**DOI:** 10.4102/sajcd.v73i1.1144

**Published:** 2026-05-26

**Authors:** Senamile G. Ntuli, Ben Sebothoma, Nabeelah Nagdee, Nicky Israel

**Affiliations:** 1 Department of Speech-Language Pathology and Audiology, School of Health Care Sciences, Sefako Makgatho Health Sciences University, Pretoria, South Africa; 2 Department of Speech Pathology and Audiology, School of Human and Community Development, Faculty of Humanities, University of the Witwatersrand, Johannesburg, South Africa; 3 Department of Psychology, School of Human and Community Development, Faculty of Humanities, University of the Witwatersrand, Johannesburg, South Africa

**Keywords:** audiologists, professional quality of life, South Africa, workplace, work–life balance, resilience, sustainability, burnout

## Abstract

**Background:**

South African audiologists face clinical and systemic challenges because of resource constraints and workload pressures, yet their professional quality of life and the role of resilience remain underexplored.

**Objectives:**

To explore the professional quality of life of South African audiologists and to examine its association with resilience and coping.

**Method:**

A two-phase explanatory sequential mixed-methods design was employed. Phase 1 involved a cross-sectional web-based survey, incorporating the Professional Quality of Life Scale (v5), while phase 2 comprised semi-structured interviews. Quantitative data were analysed using descriptive and inferential statistics, and qualitative data were analysed using Braun and Clarke’s reflexive thematic analysis.

**Results:**

Audiologists reported moderate compassion satisfaction (CS) and low burnout and secondary traumatic stress (STS). Burnout (BO) was negatively correlated with CS, while compassion fatigue (CF) was positively correlated with both BO and STS. A negative association was observed between STS and CS. Higher resilience was associated with better professional quality of life and qualitative findings highlighted emotional regulation, adaptability and support as key to sustaining professional fulfilment.

**Conclusion:**

Organisational support plays an important role in promoting audiologists’ well-being and professional engagement. Strengthening support structures and resilience practices may improve professional quality of life. There is also a need to enhance training through supervision, mentorship and the integration of resilience and coping skills into audiology curricula.

**Contribution:**

These findings can inform strategies to improve audiologists’ well-being and guide institutional and policy responses.

## Introduction

Healthcare practitioners in South Africa, including audiologists, are legally, ethically and morally obligated to provide high-quality patient care (Health Professions Council of South Africa [HPCSA], 2016). Legislation such as the *National Health Act (61 of 2003)* and ethical guidelines from the HPCSA, 2016) outline standards for confidentiality, professionalism and patient rights. However, consistently meeting these standards can be challenging, particularly in an under-resourced and high-pressure healthcare system. Audiologists face the added emotional strain of diagnosing and managing life-altering communication disorders (Gelfand, 2016), often under difficult socioeconomic conditions (Brito-Marcelino et al., 2020; Pillay et al., 2020). This can affect their professional quality of life (ProQoL).

The South African context presents challenges shared with many low- and middle-income healthcare systems that may negatively affect audiologists’ professional quality of life, such as compassion satisfaction (CS), burnout (BO) and secondary traumatic stress (STS). High crime rates, gender-based violence and frequent exposure to trauma, both directly and through patients, can contribute to emotional exhaustion and anxiety, particularly among female practitioners (Khoza-Shangase et al., 2022; Nagdee & De Andrade, 2023; Statistics South Africa, 2023). Financial stressors, such as high unemployment, rising living costs and stagnant public-sector wages, further exacerbate these pressures (Hlayisi, 2022; Moroe et al., 2025). Limited resources in many public healthcare settings often compromise audiologists’ ability to provide effective care (Maphumulo & Bhengu, 2019).

Despite the growing global interest in professional quality of life among healthcare workers (Bhutani et al., 2012; Blood et al., 2008; Chung et al., 2020; De Andrade et al., 2024; Kelly et al., 2015; Sacco et al., 2015; Severn et al., 2012; Zakeri et al., 2021; Zimmer et al., 2022), little is known about the professional quality of life of South African audiologists as conceptualised and measured using the Professional Quality of Life (ProQOL v5) scale.

This study explores factors influencing their professional quality of life through a mixed-methods approach to gain a deeper understanding of the emotional, psychological and occupational challenges faced by audiologists across diverse professional settings. Understanding the professional quality of life of South African audiologists can inform the development of interventions to enhance resilience, reduce burnout and improve job satisfaction and patient care.

Professional quality of life is both a theoretical construct and an operationalised measure encompassing both the positive and negative experiences that professionals encounter in caregiving roles. It is particularly relevant to healthcare professions, such as audiology, where empathy and emotional involvement are integral to daily practice (Moudatsou et al., 2020). Stamm (2010) defines professional quality of life as comprising two key dimensions: CS, the pleasure derived from being able to do one’s work well, and compassion fatigue (CF), the negative dimension of professional quality of life, which includes BO and STS. Burnout is associated with feelings of hopelessness, inefficacy and emotional exhaustion, while STS refers to stress resulting from exposure to others’ traumatic experiences (Bride, 2007; Stamm, 2010).

Together, BO and STS constitute CF, rather than independent components of professional quality of life. The ProQOL v5 scale was selected for this study because it provides a widely used and theoretically grounded way of assessing these dimensions in helping professionals. Understanding these dimensions is essential for supporting healthcare professionals and ensuring sustainable, high-quality service delivery.

Several studies have explored professional quality of life among various healthcare professionals, including nurses in Iran (Taghinezhad et al., 2024; Zakeri et al., 2021), nurses in the United States (Kelly et al., 2015; Sacco et al., 2015), occupational therapists in South Korea (Chung et al., 2020) and medical doctors working in government and private institutions in India (Bhutani et al., 2012). Bhutani et al. (2012) found that medical doctors working in high-pressure environments, particularly in government settings, reported lower CS and higher CF compared to those in private institutions, where better infrastructure and support systems were available. Similarly, Chung et al. (2020) reported high levels of burnout and STS among occupational therapists in South Korea, particularly among those with less support and poor working conditions. Zakeri et al. (2021) highlighted a positive relationship between CS and clinical competence among nurses, suggesting that satisfaction in one’s role contributes to higher performance and reduced burnout. Clinical experience also plays an important role in professional quality of life outcomes, with more experienced healthcare professionals generally reporting higher CS and lower levels of CF across these international contexts (Kelly et al., 2015; Sacco et al., 2015). These findings underscore the importance of work environment, institutional support and professional development in shaping professional quality of life outcomes.

### Professional quality of life of audiologists

Despite its relevance, most existing research on professional quality of life among audiologists has been conducted in high-income or developed country contexts, with comparatively little evidence available from low- and middle-income settings. In developed countries, clinical experiences have also been found to play a significant role in the professional quality of life of audiologists, with early-career audiologists exhibiting higher emotional exhaustion (Blood et al., 2008), and higher burnout and depersonalisation (Zimmer et al., 2022). This is particularly evident among audiologists working in public health sectors, serving the paediatric population (Severn et al., 2012). While these studies have provided important insight into the audiologists’ professional quality of life, the reality in low- and middle-income countries (LMICs) may be different, exacerbated by multiple factors such as systemic constraints including resource scarcity, high clinical workloads, limited infrastructure and reduced organisational support, alongside broader socioeconomic stressors common in LMIC health systems. (De Andrade et al., 2024; Masson, 2016; Nagdee & De Andrade, 2023).

### Theoretical framework

This study is guided by resilience theory, which provides a framework for understanding how individuals adapt to stress and adversity (Van Breda, 2018). Resilience is conceptualised not as a fixed trait but as a dynamic and contextually shaped process influenced by personal, relational and environmental factors that support positive adaptation (Van Breda & Theron, 2018). This lens is particularly useful for examining how audiologists navigate occupational stressors, including emotional exhaustion, workload pressures and secondary exposure to trauma.

It enables the identification of protective factors that promote well-being and support sustainable professional functioning.

From a theoretical perspective, within the context of professional quality of life, resilience is conceptualised as a critical buffer against burnout and STS, while theoretically enhancing CS. Defined as the ability to thrive amidst adversity (Gonçalves et al., 2022), resilience encompasses psychological flexibility, self-efficacy and effective coping strategies (Campbell-Sills & Stein, 2007). Research shows that resilience is among the strongest predictors of CS (Pietrzak et al., 2014), and it is often linked to increased empathy and emotional strength derived from past adversity (Lim & DeSteno, 2016). Conceptually, understanding resilience as both a buffer and a source of growth allows for deeper insight into how audiologists maintain emotional balance and professional engagement in the face of ongoing challenges.

Van Breda (2018) further distinguishes resilience as both an outcome, recovering from adversity and a process, emphasising the mechanisms that enable positive adaptation over time. This distinction is particularly relevant in audiology, where professionals face both acute (e.g. traumatic patient encounters) and chronic (e.g. prolonged workload strain) stressors. In the South African context, resilience theory highlights culturally grounded coping strategies, community support and structural challenges, while interventions may focus on developing personal resources such as self-efficacy and self-compassion (Theron, 2016; Van Breda, 2018; Van Breda & Theron, 2018).

At a theoretical and policy level, interventions that aim to strengthen personal resources such as self-efficacy and self-compassion, particularly in resource-constrained settings, are conceptualised as having the potential to enhance professional quality of life (Gonçalves et al., 2022), making resilience a key target for policy development, professional training and workplace support.

The study aimed to examine levels of professional quality of life (CS, burnout and STS) among South African audiologists, to explore associations with resilience and coping, and to gain an in-depth understanding of how these experiences are perceived and managed in practice.

### Research question

*What is the professional quality of life of South African audiologists?*

## Research methods and design

### Research design

A two-phase, sequential explanatory mixed-methods design was used (Creswell & Creswell, 2018). In the first phase, a cross-sectional web-based survey was conducted to obtain quantitative data. Phase 1 employed a non-probability convenience sampling approach, whereby eligible South African audiologists voluntarily participated by responding to an online survey distributed via professional networks and social media platforms.

Quantitative data collected in phase 1 examined levels of professional quality of life, resilience and coping, as well as associations between these constructs and selected demographic and work-related variables. The second phase involved qualitative semi-structured interviews to further explore key quantitative findings. Phase 2 employed a non-probability purposive sampling approach, drawing on phase 1 participants who indicated willingness to participate in follow-up interviews and were selected to ensure variation in years of experience and work setting.

Specifically, phase 1 results showing patterns and associations between CS, CF, resilience, coping and demographic factors (such as age and professional experience) were used to identify areas requiring deeper exploration. These quantitative patterns directly informed the focus and content of the phase 2 interview guide, allowing the qualitative data to explain how and why these relationships were experienced in practice. Thus, the quantitative phase addressed ‘what’ patterns existed in professional quality of life, while the qualitative phase sought to explain ‘how’ and ‘why’ these patterns were experienced by audiologists.

### Study setting

The sample data for this research were collected from practising audiologists across diverse work settings in South Africa, including public hospitals, community health clinics, private practices, schools and academic institutions. Participants in phase 1 were recruited using non-probability convenience sampling to obtain information on professional quality of life among audiologists. The South African healthcare context is characterised by substantial variation in resource availability, workload demands and organisational support across work settings (Maphumulo & Bhengu, 2019; Mayosi & Benatar, 2014; Pillay et al., 2020). These contextual differences may shape experiences of CS, burnout and STS among audiologists (Khoza-Shangase & Mophosho, 2018; Statistics South Africa, 2023). Accordingly, the study sought to investigate whether professional quality of life differed across work settings. Phase 2 participants were purposively selected from the phase 1 sample to allow for in-depth exploration of experiences related to professional quality of life.

### Eligibility criteria

Participants were eligible to take part in the study if they were registered audiologists with the HPCSA, currently practising in any work setting in South Africa, and proficient in English, which was the language used for the survey and interviews. Audiologists who were not currently practising, were not HPCSA-registered, or who were unable to complete the survey in English were excluded.

### Sampling

Phase 1 used a non-probability convenience sampling approach to recruit South African audiologists registered with the HPCSA and practising in any setting within the country. Invitations were distributed via professional associations and social media platforms commonly used by audiologists.

An intended sample size of 283 participants was calculated based on the estimated population of registered audiologists in South Africa, using a 95% confidence interval, a 5% margin of error and a proportion of 50% (Olowofela & Isash, 2017). A total of 96 responses were initially received. Of these, 23 responses were excluded due to substantial missing or incomplete data, resulting in a final analysed sample of 23 participants.

Phase 2 employed non-probability purposive sampling. Participants were drawn from the phase 1 survey respondents who indicated willingness to be interviewed. Selection was guided by key quantitative patterns, particularly differences in CS, burnout, resilience, age and years of experience, to ensure inclusion of diverse perspectives.

An initial target of approximately 12–20 interviews was set, consistent with recommendations for thematic saturation in qualitative health research (Braun & Clarke, 2019). Recruitment continued until data saturation was reached, defined as the point at which no new themes were emerging, which occurred after 15 interviews.

### Data collection instrument

Professional quality of life was measured using the Professional Quality of Life Scale, Version 5 (ProQOL v5), developed by Stamm (2010) to assess the positive and negative effects of caring work in helping professionals.

The phase 1 survey is provided in Online Appendix 1. Data were collected through a questionnaire that comprised informed consent information, demographic characteristics of participants and components of the ProQOL v5 scale, which consists of 3 sections and 30 items. The components of ProQOL v5 included CS, burnout and STS, with CF conceptualised as the combined impact of burnout and STS (Stamm, 2010). The components of ProQOL v5 were measured using a five-point Likert-type scale, ranging from 1 (never) to 5 (very often), with each subscale consisting of 10 items and possible scores ranging from 10 to 50. Higher scores reflect higher levels of the respective construct.

The ProQOL v5 was selected due to its widespread use in healthcare research and its established reliability and validity in assessing professional quality of life among helping professionals (Heritage et al., 2018; Sinclair et al., 2017; Stamm, 2010). The ProQOL v5 was particularly suited to this study because it captures both the positive (CS) and negative (burnout and STS) dimensions of caring work, allowing a balanced assessment of professional quality of life among audiologists working in emotionally demanding clinical contexts.

Resilience and coping were assessed using three self-report items included at the end of the survey. These items were designed to capture participants’ perceived ability to manage, recover from and adapt to work-related stress and challenges. The first item was an open-ended question: ‘How do you manage and recover from the stresses and challenges you encounter in your professional life, and how would you describe your ability to maintain your well-being despite these challenges?’ This item was analysed qualitatively. The second item assessed perceived resilience using a single Likert-type scale: ‘On a scale from 1 to 5, where one means “not at all resilient” and five means “extremely resilient,” how would you rate your resilience in managing professional stress and maintaining your well-being?’ Scores ranged from 1 to 5, with higher scores indicating greater perceived resilience. The third item assessed perceived coping using a single-item categorical scale: ‘Which of the following best describes your ability to cope with and recover from work-related stress and challenges?’ Responses ranged from ‘I often struggle to cope and feel overwhelmed’ to ‘I thrive under pressure and use challenges as opportunities for growth’, with higher categories reflecting stronger perceived coping. This item was treated as an ordinal measure of coping capacity. These single-item measures were included to minimise respondent burden in a longer survey and to allow participants to provide both quantitative and qualitative reflections on their resilience and coping.

However, unlike ProQOL, these items have not been formally psychometrically validated and should be interpreted as subjective indicators rather than standardised measures.

A semi-structured interview guide (Adeoye-Olatunde & Olenik, 2021) was developed to explain and expand on key findings from phase 1, in line with the explanatory sequential mixed-methods design. The phase 2 interview guide is provided as Online Appendix 2. The purpose of the interviews was to explore how participants experienced and interpreted patterns observed in the survey, particularly in relation to CS, CF, early-career challenges and self-reported resilience and coping. Interview questions were informed by the study aims and by phase 1 quantitative results, and were designed to elicit contextual explanations, examples and perceived drivers of professional quality of life. Quantitative findings related to resilience, coping and early-career experiences informed the development of interview questions.

A pilot study was conducted to test the feasibility of both phases (Irwin et al., 2008), involving two audiologists from different work settings. These participants completed the questionnaire and interviews and gave feedback via a structured form on clarity and relevance. They were excluded from the main study to avoid bias (Creswell, 2009).

### Data collection procedure

The ProQOL scale is freely available for use without author’s permission, provided it is not sold, credited to the Centre for Victims of Torture (www.ProQOL.org), and unaltered except for translation or replacing the word ‘helper’ with a profession-specific term (Stamm, 2009). Permission to distribute the scale online was obtained from relevant professional associations, including South African Neurodevelopmental Therapy Association (SANDTA), South African Association of Audiologists (SAAA), National Black Association for Speech-Language and Hearing (NABSLHA), and Rural Rehab South Africa (RuReSA).

In phase 1, ProQOL v5 was administered via REDCap, a secure, user-friendly online platform, after signing a user agreement. The survey link included the research invitation, phase 1 information sheet and consent form.

Participants had 3 months to complete the questionnaire, with monthly reminders sent. At the end of the survey, participants could indicate a willingness to join phase 2 and provide contact details for follow-up. Phase 2 interviews were conducted via Microsoft (MS) Teams for convenience and cost-efficiency (De Villiers et al., 2021), enabling in-depth, semi-structured discussions (Ed. Cook, 2001; Knudsen et al., 2012). Each interview lasted 30 min – 45 min (Jamshed, 2014), was audio-recorded with participant consent (Carpenter & Suto, 2008) and securely stored (Frey & Beth, 2023). A distress protocol, adapted from Draucker et al. (2009), was prepared but not activated, as no participant showed signs of distress.

Where available, participants’ phase 1 survey responses (e.g. years of experience, work setting and professional quality of life scores) were reviewed prior to the interview to support probing and clarification during the qualitative discussion, while maintaining a flexible, participant-led interview approach.

### Data processing and analysis

Before data analysis was carried out, incomplete responses from the questionnaire were reviewed and excluded from the dataset. The Statistical Package for the Social Sciences (SPSS) version 29 (IBM Corporation, 2022) was used to analyse the quantitative data. Pearson’s correlation coefficients were used to determine the strength, direction and significance of relationships between the professional quality of life subscales, resilience and coping (Puth et al., 2014). Independent sample *t* tests were conducted to compare professional quality of life scores, coping strategies and resilience levels across different participants (Kim, 2019).

In the second phase, all interviews were audio-recorded with participant consent and transcribed verbatim by the researcher. Identifying information was removed during transcription, and participants were assigned participant numbers. Digital audio files and transcripts were stored on a password-protected computer and backed up on an encrypted external drive accessible only to the research team. Reflexive thematic analysis following Braun and Clarke’s (2006) six-step approach was used. Firstly, the data were familiarised through repeated reading of the transcripts, prior to coding. Secondly, initial codes were generated, guided by both the research questions and key quantitative findings (e.g. burnout, CS, resilience and early-career vulnerability). Thirdly, codes were organised into candidate themes that aligned with major quantitative patterns, such as CS, burnout and coping. Fourthly, these themes were reviewed and refined to ensure internal coherence and clear distinction between themes. Fifthly, themes were defined and named to reflect how they explained or expanded on the quantitative results. Finally, the report was produced using NVivo fourteen to organise and retrieve data segments, with participant quotes used to illustrate how qualitative findings contextualised and deepened understanding of the quantitative trends.

In accordance with the ProQOL v5 manual (Stamm, 2010), CF was not treated as a standalone subscale but was operationalised as a derived construct, calculated by combining scores from the burnout and STS subscales. This approach reflects the conceptualisation of CF as a higher-order construct encompassing both burnout and STS. Accordingly, analyses were conducted on individual professional quality of life subscales (CS, burnout and STS), as well as on the derived CF score, to allow for both nuanced and integrative interpretation of professional quality of life outcomes. Correlation analyses were conducted to examine associations between professional quality of life variables, resilience and coping. No formal adjustment for multiple comparisons was applied, and correlations were interpreted with caution, with emphasis placed on effect sizes and consistency with theoretical expectations.

Several strategies were used to enhance the credibility and trustworthiness of the qualitative analysis. An audit trail was maintained through reflective memos and coding notes documenting analytic decisions. Preliminary themes were reviewed and discussed with co-researchers to support peer debriefing and reduce individual bias. In addition, the integration of quantitative and qualitative findings allowed for triangulation, whereby themes were examined in relation to survey patterns. Together, these strategies strengthened the credibility, dependability and transparency of the qualitative findings.

### Ethical considerations

This study was approved by the Human Research Ethics Committee (Non-Medical) of the University of the Witwatersrand, Johannesburg, South Africa (Clearance No. H24/09/38). All procedures followed the ethical principles outlined in the Declaration of Helsinki (World Medical Association, 2013), which emphasises the protection of participants’ rights, welfare and well-being. Before data collection, participants received detailed information about the study’s objectives, procedures, risks and benefits. Written informed consent was obtained from all participants. The study also followed data protection standards to ensure privacy and confidentiality throughout. Ethical approval and research design were reviewed to align with international ethical research standards (Emanuel et al., 2004; Jelsma & Clow, 2005).

## Results

Results are presented by ProQOL domain (CS, burnout, STS and CF), with quantitative findings reported first and qualitative themes used to explain and expand on each domain. The ProQOL v5 subscales have demonstrated good internal consistency reliability in the past, with original Alpha coefficients of 0.88 (CS), 0.75 (BO) and 0.81 (STS) (Stamm, 2010). In this study, Cronbach’s α coefficients were 0.90 (CS), 0.82 (BO) and 0.79 (STS). Compassion fatigue, computed as a composite of burnout and STS, demonstrated high internal consistency (Cronbach’s α = 0.86). A total of 73 South African audiologists participated. Most participants were black people (49%), predominantly female (92%) and aged 26–35 years (45%). Thirty-eight per cent of the participants had between 0 years and 5 years of working experience, while 22% had 16 years or more years of experience. The majority of the participants (63%) held a bachelor’s degree (e.g. Bachelor of Science [BSc] Audiology) as their highest qualification, with a few holding postgraduate qualifications such as a Master’s or Doctor of Philosophy (PhD). Participants were unevenly distributed across work settings, with most working in private practice (41%) or government hospitals (36%), and very few participants working in school settings (government 4%, private 3%). This pattern reflects the South African audiology labour market, in which relatively few school-based positions exist compared to hospital and private practice posts (Khoza-Shangase et al., 2022). In phase 2, 15 participants volunteered to take part in the interviews. As shown in Table 1, participants represented a range of ages, years of experience and work settings, although the sample was predominantly female and younger. The sample comprised 14 females and only one male. Nine of the participants were black African people, two were white people, while there was one Indian person and one coloured participant. There was only one participant who preferred not to disclose their ethnicity. Of the participants interviewed, eight worked in clinical settings (public or private), four in academic settings and three across mixed roles involving both clinical and academic work (Table 1). This diversity allowed exploration of how different employment contexts shaped professional quality of life, rather than equal numbers across settings.

**TABLE 1:** Demographic characteristics of Phase 1 participants.

Participant’s ID	Highest qualification	Ethnicity	Age (years)	Gender	Years of experience	Work setting
P6	Master’s	African people	36–45	Female	11–15	Government Private
P8	Doctorate	African people	46–55	Female	20+	Academic
P12	Bachelor’s	African people	18–25	Female	0–5	Private
P21	Bachelor’s	Coloured people	26–35	Female	0–5	Private
P25	Bachelor’s	White people	18–25	Female	0–5	Private
P30	Bachelor’s	Indian people	18–25	Female	0–5	Government
P33	Master’s	Indian people	26–35	Female	6–10	Academic Private
P34	Bachelor’s	African people	36–45	Female	6–10	Private
P44	Bachelor’s	African people	26–35	Female	6–10	Government
P49	Bachelor’s	African people	18–25	Female	0–5	Government
P53	Bachelor’s	African people	26–35	Female	0–5	Government
P76	Bachelor’s	White people	36–45	Female	16–0	Government
P78	Doctorate	Prefer not to say	55+	Female	20+	Academic Private Government
P82	Bachelor’s	African people	26–35	Female	0–5	Academic
P89	Doctorate	African people	26–35	Male	11–15	Government

ID, identification.

Note: Work setting refers to participants’ current employment at the time of the Phase 2 interview. Some participants reported working across multiple sectors concurrently (e.g. public hospital and private practice); therefore, categories are not mutually exclusive.

The phase 2 qualitative sample was almost entirely female and predominantly black people. While this reflects the demographic profile of the study participants, it is important to acknowledge that these characteristics may shape how professional quality of life, workplace stressors and coping strategies were experienced and articulated. In particular, participants’ perspectives are situated within broader socio-historical and structural contexts that influence professional identity, access to support and experiences of occupational strain in South Africa.

### Levels of professional quality of life and their association with demographic variables

The study assessed levels of CS and CF, comprising BO and STS, and examined their association with demographic variables in the sample.

#### Compassion satisfaction

The mean CS score was 40.18 (standard deviation [s.d.] = 7.25), indicating moderate levels overall. Older participants (≥ 36 years; *M* = 42.92) reported significantly higher CS scores (*t* = −2.33; *p* = 0.023; *d* = −0.58) than younger participants (< 36 years; *M* = 38.84). Those with more than 5 years of experience (*M* = 41.89) also had significantly higher CS levels (*t* = −2.66; *p* = 0.010; *d* = −0.64) than those with less experience (*M* = 37.43). However, no significant differences were found based on educational qualification levels (*p* > 0.05).

Qualitative interviews were used to explore how CS was experienced in practice. Qualitative analysis identified themes that explained how CS was experienced in practice, particularly through meaningful patient outcomes, professional purpose and contribution to others. For example, a participant shared, ‘That would be when I successfully complete a treatment’ (P30), while another noted the fulfilment of seeing patients benefit from care: ‘It just brings so much fulfilment because you can see this patient is benefiting from it’ (P21). A participant emphasised the rewarding nature of making a difference: ‘You can see the difference that you’re making in someone’s life’ (P25). Fulfilment also came from training future audiologists, as described by one participant: ‘I’m training future audiologists who might do great things …’ and ‘If I’m training someone to be a good audiologist, it’s going to change many patients’ lives’ (P33). Another added, ‘I can be there for students when they need me … some students are dealing with so much’ (P82).

#### Burnout

The mean burnout (BO) score among participants was 24.01 (standard deviation [s.d.] = 6.72), indicating low overall burnout, although the cut-off scores suggested that just over half of the sample (53%) fell within the moderate burnout range (Stamm, 2010). While younger audiologists and those with less experience showed slightly higher BO scores, no statistically significant differences were found across age, qualification level or years of experience (all *p* > 0.05).

Burnout emerged as a significant theme, marked by emotional exhaustion, depersonalisation and professional inefficiencies. These qualitative themes help explain the quantitative pattern of moderate burnout observed in the survey. Participants reported feeling overwhelmed, fatigued, and in some cases, described thoughts of leaving the profession. Emotional exhaustion stemmed from high workloads, understaffing and lack of support.

Depersonalisation was expressed through emotional detachment and empathy fatigue due to repeated, demanding patient interactions. Professional inefficiencies, driven by resource shortages, limited growth and inadequate compensation, led to frustration and perceived underperformance. Collectively, these experiences reflect a profession under strain, with implications for both audiologists’ well-being and perceived quality of care (see Table 2 for participant quotes).

**TABLE 2:** Participant quotes related to burnout sub-themes.

Sub-themes	Interview excerpts
Emotional exhaustion	‘[*L*]ast year I was very burnt out and I was very, you know, frustrated didn’t want to go to work anymore. The stress is high.’ (P25, Mid-career audiologist, public hospital) ‘I feel when I just started off you know how it is when you’ve just like started off comm serve. You want to do everything you excited for everything but like now towards the end it’s become a bit more like I just feel like I’m really tired all the time.’ (P30, Early-career audiologist, public hospital)
Depersonalisation	‘[*W*]e have quite a high patient, quite a high outpatient load. I have to put them on the waiting list again or you know like I tend to yeah and then detachment I guess I do also feel that also.’ (P30, Early-career audiologist, public hospital) ‘Oh where you actually get empathy fatigue where you actually start not empathising with people because you empathise so much.’ (P76, Senior audiologist, mixed practice)
Professional inefficiencies	‘So with government, why I say it’s extremely stressful is because of the lack of resources. The shortage of hearing aids was challenging. It was prohibiting me from performing my best because of the difficulty in accessing the resources.’ (P33, Mid-career audiologist, public hospital) ‘[*T*]he lack of resources prevents me from being the best at my job professionally.’ (P89, Early-career audiologist, public hospital)

#### Secondary traumatic stress

Participants reported a low overall level of STS, with a mean score of 22.27 (s.d. = 6.37). While slightly higher STS scores were observed among older audiologists and those with less than 5 years of experience, none of the differences across age, qualification or experience were statistically significant (*p* > 0.05).

Qualitative themes were used to explain how STS was experienced despite relatively low average STS scores in the survey. Secondary traumatic stress emerged through the emotional burden audiologists reported experiencing when witnessing patient suffering. Participants described feeling deep emotional distress, especially when working with vulnerable groups like children. A participant noted, ‘The most emotionally challenging because you become so invested … because they’re children’ (P76). Limited resources heightened this strain, as audiologists struggled with the inability to provide adequate care. As one explained, ‘Sometimes you don’t have the equipment or the supplies that you need in order to help everyone’ (P53). Several reflected on the emotional toll of hearing both patients’ and students’ difficult experiences. A participant shared ‘Even if you try not to, you just feel for them’ (P30), while another stated ‘I’ve heard some pretty horrendous things … that have been difficult for me emotionally’ (P78). These insights highlight the emotional impact of audiological work and the pressing need for support systems and coping strategies to manage STS.

#### Compassion fatigue

Participants reported a moderate level of CF, with a mean score of 46.29 (s.d. = 11.19). While slightly higher CF scores were observed among older participants and those with less than 5 years of experience, none of these differences, across age, qualification or experience, were statistically significant (*p* > 0.05). Qualitative themes were used to contextualise the moderate CF scores observed in phase 1.

Qualitative findings further contextualised these results, revealing that CF among audiologists stemmed from both emotional and systemic stressors. Physical and emotional exhaustion was commonly linked to challenging patient interactions. As a participant explained:

‘It does cause a bit more stress because sometimes if the patient is a bit difficult, you obviously have to deal with them, be patient on your part like you are the professional.’ (P30, Bachelor’s, Indian person)

Additional stressors included financial and logistical constraints. For example, P6 cited ‘budget constraints’ and ‘time constraints’ as factors that compounded daily pressures.

Empathy overload also emerged as a key contributor to CF. Empathy overload refers to feeling emotionally overwhelmed after repeatedly caring for people in distress, which can lead to exhaustion and emotional withdrawal (Decety & Lamm, 2009). A participant reflected, ‘They tend to get empathy fatigue and then just stagnate, and they don’t try and better themselves’ (P76), illustrating the emotional cost of continuous exposure to patient suffering. Structural challenges, such as long waiting lists and understaffing, intensified these experiences. Another stated, ‘It’s just overwhelming because we have long waiting lists and we are short staffed’ (P34). Moral distress was another dimension of CF, particularly when participants were unable to provide timely or adequate care. A participant shared:

‘Then it becomes a little not so satisfying because it’s a case of we are seeing them, and then we tell them, since this is a public hospital, we have to place you onto the waiting list.’ (P30, Bachelor’s, Indian person).

Limited access to essential resources also contributed to a sense of professional inadequacy. As a participant explained, ‘It was prohibiting me from performing my best because of the difficulty in accessing the resources’ (P33). These findings underscore the complex interplay between emotional demands, resource constraints and organisational systems in shaping CF among South African audiologists.

### Relationship between the professional quality of life variables

The results revealed significant associations among the professional quality of life variables in the sample. A strong, significant, negative correlation was found between burnout and CS (*r* = −0.73; *p* < 0.001), indicating that higher burnout was linked to lower CS. Compassion fatigue also showed a moderate, significant, negative correlation with CS (*r* = −0.57; *p* < 0.001). An unexpected weak and non-significant negative correlation was observed between STS and CS (*r* = −0.22; *p* = 0.057). Secondary traumatic stress was moderately, significantly and positively correlated with burnout (*r* = 0.46; *p* < 0.001).

Correlation analyses examined relationships among ProQOL domains and self-reported resilience and coping (Table 3). Resilience showed a moderate, significant, positive correlation with CS (*r* = 0.43; *p* < 0.001) and moderate, significant, negative correlations with CF (*r* = −0.56; *p* < 0.001), burnout (*r* = −0.47; *p* < 0.001) and STS (*r* = −0.48; *p* < 0.001). In this study, resilience was quantitatively operationalised using a single self-report item included in the phase 1 survey, in which participants rated their perceived ability to manage professional stress on a five-point Likert scale (1 = not at all resilient to 5 = extremely resilient). This item captured subjective perceived resilience rather than a standardised psychometric construct and was therefore interpreted as an indicator of participants’ self-appraised coping capacity, rather than a validated resilience score. Similarly, coping was moderately, significantly and positively correlated with CS (*r* = 0.41; *p* < 0.001) but showed significant, negative correlations with CF (*r* = −0.54), burnout (*r* = −0.47; *p* < 0.001) and STS (*r* = −0.45; *p* < 0.001) (Table 3). These associations indicate that higher self-reported resilience and coping were linked to more favourable professional quality of life scores, although these relationships should be interpreted as correlational rather than causal.

**TABLE 3:** The correlations between compassion satisfaction, burnout and secondary traumatic stress, compassion fatigue, resilience and coping scores.

Variable Pair (comparison)	Pearson correlation	Sig. (2-tailed)	95% confidence intervals (two-tailed)†	
			Lower	Upper
CS – BO	-0.733	< 0.001	-0.824	-0.605
CS – STS	-0.223	0.057	-0.431	-0.007
CS – CF	-0.567	< 0.001	-0.705	-0.388
CS – RES	0.429	< 0.001	0.221	0.600
CS – CPE	0.414	< 0.001	0.203	0.588
BO – STS	0.460	< 0.001	0.258	0.624
BO – CF	0.863	< 0.001	0.789	0.912
BO – RES	-0.471	< 0.001	-0.633	-0.270
BO – CPE	-0.473	< 0.001	-0.634	-0.272
STS – CF	0.846	< 0.001	0.765	0.901
STS – RES	-0.480	< 0.001	-0.640	-0.281
STS – CPE	-0.454	< 0.001	-0.620	-0.250
CF – RES	-0.556	< 0.001	-0.697	-0.374
CF – CPE	-0.543	< 0.001	-0.687	-0.357
RES – CPE	0.692	< 0.001	0.549	0.795

CS, compassion satisfaction; CF, compassion fatigue; STS, secondary traumatic stress; RES, resilience; CPE, coping; Sig., significance.

† , Estimation is based on Fisher’s r-to-z transformation.

Qualitative findings were then used to interpret these relationships by illustrating how participants experienced resilience, coping and emotional strain in their work.

### Resilience and professional quality of life

The following section integrates quantitative associations with qualitative descriptions of resilience. Participants demonstrated moderate to high levels of resilience (*M* = 3.81, s.d. = 0.87), with slightly higher scores among older audiologists and those with more than 5 years of experience; however, these differences were not statistically significant across age, qualification, or experience groups (*p* > 0.05). Qualitative findings highlighted resilience as an important self-perceived capacity associated with more favourable professional quality of life experiences, shaped by emotional regulation, adaptability, problem-solving and strong collegial support. Qualitative findings were used to interpret the associations between self-reported resilience and professional quality of life observed in the survey. Audiologists described perseverance and composure in the face of stress, with a participant defining resilience as ‘the ability to go on and it’s the ability to bounce back’ (P25), and another noting, ‘You can’t get angry with the patient or … let them see that it’s affecting you’ (P30). Participants also demonstrated adaptability in navigating job uncertainty and resource limitations, as seen in P12’s and P49’s proactive problem-solving efforts. Support systems played a vital role, with several participants, such as P44 and P76, emphasising the importance of teamwork and shared resilience. These findings suggest that resilience supports audiologists’ professional well-being and enables them to continue providing care despite systemic and emotional challenges. These findings suggest that perceived resilience was linked to how audiologists experienced and managed their professional challenges, rather than implying a causal protective effect.

To further interpret the quantitative finding that resilience was positively associated with CS and negatively associated with burnout and CF, qualitative analysis revealed several self-reported strategies used by participants, including self-care practices, emotional regulation and professional boundary setting.

### Resources and strategies supporting professional quality of life: Qualitative themes

This section presents qualitative findings from phase 2 that were used to explain and expand on the quantitative associations observed in phase 1 between professional quality of life, resilience and coping. Interview data were analysed to identify the resources and strategies audiologists described as supporting or constraining their ability to manage stress, maintain CS and cope with burnout and STS.

#### Theme 1: Managerial and organisational support

Audiologists identified managerial support and access to mental health services as key resources for improving their professional quality of life. This theme helps explain the quantitative finding that higher coping and resilience were associated with more favourable professional quality of life outcomes. Supportive managers, who offer guidance, recognise signs of distress, and create safe spaces for open communication were seen as crucial for coping with workplace stress. As a participant noted, ‘She can help me with what I’m supposed to do … that reassures me’, and ‘She started picking up when I just can’t with a patient anymore, and she’ll jump in’ (P21). Another participant similarly valued having a manager they could approach with concerns: ‘I always go to my line manager and say this is not working’ (P33).

#### Theme 2: Access to psychological and counselling services

In addition to leadership, institutional counselling services were acknowledged as important but often insufficient.

Participants raised concerns about limited access and long waiting times, which reduced the effectiveness of support. A participant stated, ‘There’s only two therapists … you’ll only be seen within four months’ (P53), highlighting delays that hinder timely mental healthcare. While some departments were described as accommodating and understanding, consistent access to psychological services remained a concern. As one participant explained, ‘It’s an institutional counselling and support kind of service … about eight sessions with a psychologist’ (P78). These findings illustrate how organisational resources can either strengthen or constrain audiologists’ capacity to cope with emotional and occupational demands.

#### Theme 3: Personal coping and self-care strategies

Participants reported a moderate to high level of self-reported coping (CPE), with a mean score of 3.33 (s.d. = 1.07). While slightly higher CPE scores were noted among younger audiologists and those with more experience, no statistically significant differences were observed across age, qualification, or experience groups (*p* > 0.05). The qualitative data were used to explain what ‘coping’ meant in practice for participants.

Self-care practices such as rest and physical activity were commonly mentioned. A participant stated, ‘Normally, I’d try to sleep’ (P12), and another noted, ‘I think I manage it with exercise’ P(76). Work-life boundaries were also essential, as a participant explained, ‘I don’t take stuff home with me in my head’ (P78), and one participant added, ‘I’m very careful not to let work issues … consume me’ (P6). Spirituality and mental healthcare also supported emotional well-being. A participant shared, ‘I’m a Christian. So I pray …’ (P12), while others, sought therapy or medical treatment: ‘I’ve been in therapy for a while’ (P21) and ‘I’ve now been on an antidepressant … it’s just a game changer’ (P25).

#### Theme 4: Collegial and social support

Support systems, including family, friends and colleagues, were crucial. A participant mentioned, ‘Spending time with loved ones … helps me cope’ (P30), and another said, ‘I speak to my fellow colleagues and friends’ (P34). Positive team dynamics and feedback from colleagues also contributed to maintaining morale. One participant valued peer reassurance: ‘They can reassure me that … the way you did this is good …’ (P21). Flexibility and autonomy in managing workloads were highlighted as protective strategies, with a participant stating, ‘I can choose whether I want to see a lot of patients … or just take it as an easy day’ (P21). Participants also advocated for stronger managerial support and communication, with one recommending, ‘implement strategies to train the managers …’ (P34).

Together, these findings show that the single-item coping measure captured participants’ subjective perceptions of their ability to manage work stress, while the interviews revealed the concrete behaviours, resources and strategies underlying those ratings.

## Discussion

This study explored the professional quality of life of South African audiologists, revealing moderate levels of CS and CF, comprising of BO and STS. While these findings are generally consistent with previous studies (De Andrade et al., 2024; Zimmer et al., 2022), they also reflect the South African context, which shares many features with other resource-constrained healthcare systems in LMICs, including high patient loads, limited human and material resources, administrative inefficiencies and persistent structural inequalities (Khoza-Shangase et al., 2022; Maphumulo & Bhengu, 2019; Phanguphangu et al., 2024; Pillay et al., 2020), which are shaped in part by historical inequalities rooted in apartheid that continue to influence patterns of resource distribution, service access and workforce deployment within the South African health system, all which shape how audiologists experience their work (Khoza-Shangase & Mophosho, 2018; Sebothoma & Khoza-Shangase, 2021). Throughout this discussion, findings are interpreted within this broader systemic context, rather than as isolated individual-level experiences.

Compassion satisfaction was positively associated with age and experience, suggesting that professional maturity may enhance job fulfilment, likely due to increased confidence, refined clinical skills and more established coping strategies. However, younger and early-career audiologists reported lower CS and higher levels of burnout, underscoring the need for early-career support, mentorship and professional development opportunities. These findings align with previous research indicating that professional maturity, often gained through years of experience, contributes to increased job satisfaction, emotional resilience and the development of effective coping strategies (Bhutani et al., 2012; Kelly et al., 2015; Sacco et al., 2015). Less experienced professionals may be more vulnerable to emotional exhaustion due to limited exposure to challenging clinical environments and fewer established mechanisms for managing stress (Coetzee & Laschinger, 2018; RuizFernández et al., 2021).

This is particularly relevant in the South African healthcare context, characterised by historically rooted inequalities in resource distribution and workforce deployment, where early-career audiologists, often deployed to rural or under-resourced facilities for community service (Khoza-Shangase et al., 2022; Moroe et al., 2025), are expected to function independently with minimal guidance, overwhelming caseloads and insufficient equipment (Maphumulo & Bhengu, 2019; Moroe et al., 2025; Phanguphangu et al., 2024; Sebothoma & Khoza-Shangase, 2021).

These realities heighten their susceptibility to burnout and emotional exhaustion and may undermine opportunities for developing professional satisfaction and confidence. Burnout and CF were significantly and negatively correlated with CS, reinforcing the inverse relationship between emotional exhaustion and professional fulfilment, as documented in prior research (Maslach & Leiter, 2008; Stamm, 2010). Similarly, the qualitative data expand on the quantitative trend, indicating higher burnout and CF among younger audiologists by providing contextual insight into transitional stressors, supervision gaps and role overload during early professional development.

These findings highlight important implications for professional preparation and support structures. Enhanced community service preparation, clearer supervision models and structured mentorship may help mitigate early-career distress. Efforts to strengthen managerial support, supervision quality, access to counselling services and collegial support structures are therefore important for sustaining CS and long-term professional engagement among audiologists. In South Africa’s public sector, where staffing shortages, high patient volumes and limited psychological support are the norm, such interventions are especially necessary (Maphumulo & Bhengu, 2019; Moroe et al., 2025; Pillay et al., 2020; Sebothoma & Khoza-Shangase, 2021). Without them, the chronic emotional demands placed on audiologists are likely to persist, threatening retention and service quality in the profession.

Resilience was significantly associated with greater CS and lower CF (BO and STS), indicating a pattern consistent with a potential buffering role described in resilience theory, although causal relationships cannot be inferred from these data. The qualitative findings help explain the observed quantitative association between self-reported resilience and CS by showing how managerial support, access to counselling services, personal coping strategies and collegial relationships enabled participants to maintain meaning and professional fulfilment despite high emotional demands. Audiologists described resilience as emerging from emotional regulation, adaptability, problem-solving and access to organisational and social support systems, including managers, colleagues and mental health services, confirming the central role of both personal and environmental factors in maintaining psychological well-being (Coetzee & Laschinger, 2018; Van Breda & Theron, 2018). At an educational level, these findings support the inclusion of self-care, coping and resilience-building skills within undergraduate and postgraduate audiology curricula, alongside technical and clinical competencies.

A weak and unexpected relationship between STS and CS was observed, contradicting earlier findings (Nagdee & De Andrade, 2023; Severn et al., 2012). Based on prior research, a modest inverse relationship between STS and CS might have been anticipated, as higher exposure to secondary trauma has often been associated with reduced professional fulfilment in helping professions (e.g. Craig & Sprang, 2010; Stamm, 2010). The absence of a significant association in this study may reflect contextual factors specific to South African audiology practice. Audiologists may simultaneously experience exposure to emotionally demanding clinical encounters while maintaining a strong sense of meaning and purpose derived from patient impact, professional identity and community contribution. In resource-constrained settings, CS may therefore coexist with STS rather than being directly diminished by it.

Although STS was relatively low, qualitative data suggested that emotional strain, moral distress and systemic barriers (e.g. long waiting lists, insufficient resources) still affect audiologists’ mental health. These findings are consistent with research indicating that healthcare professionals in under-resourced settings often experience moral distress when unable to provide adequate care due to structural limitations (Nagdee & De Andrade, 2023; Severn et al., 2012).

While intersectional influences related to race, class and geography are critical to understanding professional quality of life in South Africa, the present quantitative dataset did not support detailed intersectional or subgroup analyses due to the limited sample size and uneven distribution across demographic and work setting categories. As such, references to intersectionality in this discussion are intended to provide contextual insight rather than empirical comparison.

Importantly, this study highlights how professional quality of life among audiologists is shaped by the interaction between emotional labour, organisational conditions and access to supportive resources within resource-constrained healthcare systems. By demonstrating how CS, burnout and STS are linked to managerial support, counselling access, personal coping strategies and collegial relationships, this research provides actionable insight into where interventions may be most effective. These findings offer a meaningful foundation for strengthening workforce support, supervision and mental health provision for audiologists working in demanding clinical environments.

### Limitations

This study has several limitations that should be considered when interpreting the findings. Firstly, although an intended quantitative sample size of 283 participants was calculated, the sample achieved comprised only 73 participants. This reduced sample size may have limited statistical power, particularly for subgroup comparisons across demographic variables and work settings and may have increased the risk of Type II errors in correlation analyses. As a result, it was not possible to conduct robust comparisons between private-sector and public-sector audiologists, despite the work setting being included as a study variable. Consequently, non-significant findings should be interpreted with caution, and future studies with larger and more evenly distributed samples are needed to examine organisational differences in professional quality of life more rigorously.

Secondly, the use of non-probability convenience sampling in phase 1 may have introduced self-selection bias, particularly as recruitment was conducted via professional associations, professional networks and social media platforms, which may have attracted audiologists with stronger views or experiences related to their professional quality of life.

Thirdly, the sample was predominantly female, younger and concentrated in private practice and public hospital settings. While this distribution reflects known trends within the South African audiology workforce, it may limit the generalisability of the findings to male audiologists, older practitioners and those working in underrepresented settings such as schools or rural community clinics.

Fourthly, although the HPCSA maintains registry data on registered audiologists, there is no single, comprehensive, publicly accessible dataset that provides detailed and directly comparable demographic information (e.g. age distribution, work setting and geographic location) for the audiology workforce at the time of the study. As a result, it was not possible to formally assess whether the achieved sample differed systematically from the broader audiologist population in South Africa.

Fifthly, while the ProQOL scale has demonstrated good psychometric properties internationally, there is limited published evidence regarding its psychometric performance specifically within South African audiologists. In addition, resilience and coping were assessed using single-item self-report indicators rather than standardised multi-item psychometric scales. While such items can capture global self-perceptions and reduce respondent burden, they do not allow for the same level of psychometric evaluation and may be more susceptible to measurement error. The absence of a comprehensive local psychometric evaluation may therefore affect the precision with which these constructs were measured in this sample.

Sixthly, given the number of correlation analyses conducted, the absence of formal adjustment for multiple testing increases the possibility that some smaller or marginally significant associations may be unstable or reflect Type I error. Findings should therefore be interpreted cautiously, particularly where effect sizes were small.

Finally, the cross-sectional design and reliance on self-report measures limit the ability to draw causal inferences and may be subject to response and recall bias. Despite these limitations, the mixed-methods design strengthened the study by allowing quantitative findings to be contextualised through rich qualitative data, enhancing the depth and credibility of the conclusions.

## Conclusion

This study addressed the critical issue of professional quality of life among South African audiologists, focusing on CS, CF, burnout and STS. The research aimed to examine how demographic variables, resilience and coping mechanisms influenced professional quality of life in a profession that remains underexplored in this context.

The findings indicate that South African audiologists in this sample experienced moderate levels of CS and CF, alongside relatively low levels of burnout and STS.

Younger and early-career audiologists appeared more vulnerable to emotional exhaustion, while resilience was associated with higher CS and lower levels of professional distress, suggesting an important relational pattern rather than a causal effect.

The qualitative findings extended these results by showing that professional quality of life was shaped by four interrelated supports: managerial and organisational leadership, access to psychological and counselling services, personal coping and self-care strategies and collegial and social support. Together, these factors influenced how audiologists managed emotional demands, sustained professional meaning and coped with structural and workload pressures.

These findings underscore the need for targeted institutional interventions, particularly in high-demand and resource-constrained healthcare settings. Strengthening supervisory structures, improving access to mental health services, investing in leadership development, and fostering supportive team cultures may be especially beneficial for early-career audiologists. However, financial and staffing constraints in many public health systems may limit the immediate feasibility of some interventions, highlighting the need for scalable, low-cost approaches such as online resilience training, peer-support platforms and self-guided self-care resources.

Future research would benefit from longitudinal designs to examine how professional quality of life, resilience and coping evolve across career stages, particularly during community service and early professional practice.

Further studies should also explore differences across work settings, including public and private sectors, to better understand how organisational context shapes professional quality of life. In addition, intervention studies evaluating structured supervision programmes, mentorship initiatives and curriculum-based resilience and self-care training would provide valuable evidence on strategies to reduce burnout and support sustainable careers in audiology.
